# Annexin A11 aggregation in FTLD–TDP type C and related neurodegenerative disease proteinopathies

**DOI:** 10.1007/s00401-024-02753-7

**Published:** 2024-06-19

**Authors:** John L. Robinson, EunRan Suh, Yan Xu, Howard I. Hurtig, Lauren Elman, Corey T. McMillan, David J. Irwin, Sílvia Porta, Vivianna M. Van Deerlin, Edward B. Lee

**Affiliations:** 1 Center for Neurodegenerative Disease Research, Department of Pathology and Laboratory Medicine, Institute On Aging, Perelman School of Medicine, University of Pennsylvania, 613A Stellar Chance Laboratories, 422 Curie Blvd, Philadelphia, PA 19104 USA; 2 Department of Neurology, Perelman School of Medicine, University of Pennsylvania, Philadelphia, PA USA

**Keywords:** Neurodegenerative disease, TDP-43, FTLD–TDP, ALS, Annexin A11

## Abstract

TAR DNA-binding protein 43 (TDP-43) is an RNA binding protein found within ribonucleoprotein granules tethered to lysosomes via annexin A11. TDP-43 protein forms inclusions in many neurodegenerative diseases including amyotrophic lateral sclerosis (ALS), frontotemporal lobar degeneration with TDP-43 inclusions (FTLD–TDP) and limbic predominant age-related TDP-43 encephalopathy neuropathologic change (LATE-NC). Annexin A11 is also known to form aggregates in ALS cases with pathogenic variants in *ANXA11*. Annexin A11 aggregation has not been described in sporadic ALS, FTLD–TDP or LATE-NC cases. To explore the relationship between TDP-43 and annexin A11, genetic analysis of 822 autopsy cases was performed to identify rare *ANXA11* variants. In addition, an immunohistochemical study of 368 autopsy cases was performed to identify annexin A11 aggregates. Insoluble annexin A11 aggregates which colocalize with TDP-43 inclusions were present in all FTLD–TDP Type C cases. Annexin A11 inclusions were also seen in a small proportion (3–6%) of sporadic and genetic forms of FTLD–TDP types A and B, ALS, and LATE-NC. In addition, we confirm the comingling of annexin A11 and TDP-43 aggregates in an ALS case with the pathogenic *ANXA11* p.G38R variant. Finally, we found abundant annexin A11 inclusions as the primary pathologic finding in a case of progressive supranuclear palsy-like frontotemporal dementia with prominent striatal vacuolization due to a novel variant, *ANXA11* p.P75S. By immunoblot, FTLD–TDP with annexinopathy and *ANXA11* variant cases show accumulation of insoluble ANXA11 including a truncated fragment. These results indicate that annexin A11 forms a diverse and heterogeneous range of aggregates in both sporadic and genetic forms of TDP-43 proteinopathies. In addition, the finding of a primary vacuolar annexinopathy due to *ANXA11* p.P75S suggests that annexin A11 aggregation is sufficient to cause neurodegeneration.

**Supplementary Information:**

The online version contains supplementary material available at 10.1007/s00401-024-02753-7.

## Introduction

TAR DNA-binding protein 43 (TDP-43) is predominantly found in the nucleus where it regulates splicing. However, a fraction of TDP-43 is found outside the nucleus where, along with G3BP1 and other proteins, it is found in cytoplasmic and neuritic RNP granules [20]. In neurons, RNP granules can be tethered to lysosomes by annexin A11, allowing for their transport along axons or dendrites, presumably for localized translation [2, 3]. In the Center for Neurodegenerative Disease Research (CNDR) brain bank, TDP-43 accumulates as a major disease causing proteinopathy in 88% of amyotrophic lateral sclerosis (ALS) patients, 55% of late-onset Alzheimer’s disease (AD) patients where it is described as limbic-predominant age-related TDP-43 encephalopathy neuropathologic change (LATE-NC), and 42% of frontotemporal degeneration (FTD) patients where it is known as frontotemporal lobar degeneration with TDP-43 inclusions (FTLD–TDP). FTLD–TDP pathology shows diverse histological patterns, or subtypes, that correlate with clinical phenotypes, different genetic causes, and distinct atrophy patterns [13, 16]. The most common subtypes are types A, B and C.

FTLD–TDP Type C is typically sporadic and often manifest as semantic variant of primary progressive aphasia with prominent temporal polar atrophy [7, 22, 28]. Microscopically, Type C TDP-43 aggregates are primarily composed of long and thick dystrophic neurites (DNs) throughout cortical layers with relatively few cortical neuronal cytoplasmic inclusions (NCIs) but abundant compact NCIs in the dentate gyrus and striatum [16]. In contrast, Type A pathology is associated with pathogenic *GRN* variants and typically manifest as either behavioral variant of FTD or nonfluent/agrammatic primary progressive aphasia, exhibiting more dorsal frontal and temporal atrophy. Type B cases are often associated with *C9orf72* repeat expansions and typically manifest as behavioral variant of FTD with or without motor neuron disease, often with relatively symmetric, frontoinsular and thalamic atrophy.

Several studies have reported *ANXA11* variants associated with ALS [17, 25, 26]. The *ANXA11* gene encodes annexin A11, a member of the annexin family of calcium-dependent phospholipid-binding proteins involved in a variety of cellular functions including mRNA transport and translation, endocytosis, exocytosis, and plasma membrane repair [10]. One pathogenic variant, *ANXA11* p.D40G, found in cases of familial ALS has been described as resulting in unique skein-like or basket-like annexin A11 aggregates which are distinct from and do not co-localize with TDP-43 aggregates [25]. In contrast, ALS due to *ANXA11* p.G38R and c.1086 + 1G > A has been associated with annexin A11 aggregates in the form of NCIs, DNs and skein-like inclusions which comingle with TDP-43 aggregates in cortical, hippocampal, subcortical and spinal cord neurons [23, 26]. Annexin A11 pathology has not been described in sporadic ALS, FTLD–TDP or LATE-NC cases.

In addition to forming protein aggregates in cases carrying rare *ANXA11* variants [18, 25], TDP-43 and annexin A11 share common biochemical features including their ability to undergo liquid–liquid phase separation via relatively weak but multi-valent interactions mediated by their low-complexity domains [11]. Moreover, TDP-43 can be found within axonal RNP transport granules where annexin A11 tethers these RNP granules to lysosomes for long distance trafficking and delivery to distal locations within neurons [1, 14].

To explore the relationship between TDP-43 and annexin A11 in neurodegenerative diseases, we first performed a genetic analysis to identify rare *ANXA11* variants in a cohort of 822 CNDR autopsies, followed by immunohistochemistry to assess for the presence of annexin A11 aggregate pathology in the cases carrying the rare *ANXA11* variants. We then extended our immunohistochemistry study to screen a wide variety of sporadic and genetic forms of ALS, FTLD–TDP, LATE-NC and other neurodegenerative disease cases for annexin A11 aggregates. Our major finding is the presence of insoluble annexin A11 aggregates in all FTLD–TDP Type C cases. We show that TDP-43 and annexin A11 pathology typically have similar morphology and regional prevalence in FTLD–TDP Type C. Annexin A11 inclusions were also seen in a small proportion of LATE-NC, FTLD–TDP Type A and B, and ALS cases. We also corroborate the co-localization of annexin A11 and TDP-43 aggregates in a case of ALS due to *ANXA11* p.G38R. Finally, we report a novel *ANXA11* p.P75S variant in a case of progressive supranuclear palsy-like FTD syndrome, previously diagnosed neuropathologically as frontotemporal lobar degeneration with TDP-43, tau and alpha-synuclein-negative, ubiquitin-positive inclusions (FTLD-U). Autopsy revealed prominent striatal vacuolization in association with annexin A11 aggregates which appeared to be the primary pathologic inclusion.

## Materials and methods

### Genetic screen

*ANXA11* genetic variants in 822 neuropathologically diagnosed CNDR cases (Supplemental Table 1) were identified from whole genome sequencing, whole exome sequencing, or targeted neurodegenerative disease sequencing panel datasets [27]. Variant data was imported and analyzed in Geneticist Assistant interpretative workbench (SoftGenetics). Rare variants with a minor allele frequency (MAF) < 0.05% (gnomAD v.3) were selected and in silico tools including CADD, REVEL, SIFT, PolyPhen, MutationTaster, and MutationAssessor were used to classify potential pathogenicity. The screen yielded four variants of uncertain significance and a previously reported familial ALS p.G38R variant (Table 1) [25, 26]. All five rare *ANXA11* variants were confirmed by Sanger sequencing.

**Table 1:** Clinicopathological data of rare *ANXA11* variants

Case	*ANXA11* variant	Pathogenicity	GnomAD	Annexin A11 inclusions	Clinical diagnosis	Onset (y)	Age (y)	Sex	Neuropathological diagnosis
1	c.223C > T, p.P75S	Uncertain significance	Absent	Yes	PSP/FTD-NOS	65–70	70–75	M	FTLD-U
2	c.112G > A, p.G38R	Pathogenic	4.60E-05	Yes	ALS (Definite)	60–65	60–65	M	ALS
3	c.595G > A, p.G199S	Uncertain significance	8.55E-05	Yes	bvFTD	60–65	80–85	M	FTLD–TDP
4	c. 1423C > T, p.R475W	Uncertain significance	7.90E-05	No	PSP	60–65	60–65	F	PSP
5	c.1010 T > A, p.L337H	Uncertain significance	2.30E-04	No	PSP	60–65	70–75	M	PSP

*AD* Alzheimer’s disease, *ALS* Amyotrophic Lateral Sclerosis, *bvFTD* behavioral variant Frontotemporal degeneration, *FTD-NOS* Frontotemporal degeneration not otherwise specified, *FTLD-U* Frontotemporal Lobar Degeneration with ubiquitin positive inclusions, *PSP* progressive supranuclear palsy. Age ranges are provided to help maintain anonymity

### Immunohistochemistry

All five *ANXA11* variant cases were stained using immunohistochemistry to assess for the presence of annexin A11 aggregates using an anti-annexin A11 antibody (Proteintech #1A3C4, mouse monoclonal, 1:8,000). In addition, an immunohistochemical screen for annexin A11 aggregate pathology was performed with the same antibody to assess for the presence of annexin A11 aggregates in ALS, FTLD–TDP, LATE-NC and other neurodegenerative disease cases (n = 368 cases including the *ANXA11* variant cases, Supplementary Table 2). Briefly, sections were subject to microwave antigen retrieval, stained with primary antibody overnight followed by biotinylated secondary antibodies, amplified using an ABC kit (Vectorlabs), and visualized using immPACT DAB (Vectorlabs). Regions screened involved regions of TDP-43 pathology including motor cortex, spinal cord, and medial temporal lobe (amygdala or hippocampus) in ALS cases, frontal or temporal cortex and medial temporal lobe in FTLD–TDP cases, and medial temporal lobe in LATE-NC cases. Annexin A11 pathology was confirmed with a second annexin A11 antibody (Abcam #ab236599, rabbit monoclonal, 1:4,000) for a subset of cases which showed similar results. Additional immunohistochemical stains were performed using antibodies that recognize phosphorylated TDP-43 (pTDP-43; 1D3, 1:300, a gift of Elisabeth Kremmer and Manuela Neumann), FUS (Proteintech 60,160-1-Ig, mouse monoclonal, 1:8,000) or G3BP1 (Proteintech 13,057–2-AP, rabbit polyclonal, 1:1,000). Immunohistochemistry images were captured on a Nikon Eclipse Ni microscope.

Immunofluorescence was performed with an anti-annexin A11 rabbit polyclonal antibody (Proteintech #10,479-2-AP, 1:4,000) together with an anti-ubiquitin mouse monoclonal (Millipore #1510, 1:20,000), or an anti-annexin A11 mouse monoclonal (Proteintech #1A3C4, 1:4,000) together with a pTDP-43 antibody (1D3, 1:150), all with microwave antigen retrieval, and visualized with fluorescently labelled secondary antibodies (Invitrogen Alexa Fluor 488 and Alexa Fluor 594). Immunofluorescence images were obtained using a Leica TCS SPE laser scanning confocal microscope.

### Protein biochemistry

Human post-mortem brain tissue was subject to sequential extraction using buffers of increasing strength as previously described [19]. Frontal or amygdalar region (amygdala with periamygdalar cortex) was used for FTLD–TDP cases and anterior cingulate cortex was used for the *ANXA11* variant cases. Briefly, grey matter samples were first extracted with 1% Triton X-100 high salt buffer, then treated with Benzonase after myelin removal and extracted with 2% sarkosyl high salt buffer. The pellet was washed and re-suspended with Dulbecco’s phosphate-buffered saline by sonication using a hand-held probe (QSonica, Newtown, CT) before a final spin at 5000 × g for 5 min at 4 °C was performed to remove large protein debris and to obtain the final sarkosyl-insoluble fraction. Total protein concentration was measured by BCA assay (Thermo Scientific Inc., Rockford, IL). The annexin A11 positive control consisted of RIPA soluble extract from QBI293 cells (iGFP-NLSm) [19]. The fractions were analyzed on 12% Bis–Tris gels (NuPAGE Novex) and nitrocellulose membranes were immunoblotted with two antibodies and visualized with IRDye-labelled secondary antibodies using an Odyssey ODY-2816 Imager and LI-COR Image Studio software. The annexin A11 bands were initially analyzed using two ANXA11 antibodies (Proteintech #1A3C4 at 1:10,000 and Abcam #ab236599 at 1:1,000) (Supplementary Fig. 1). A second immunoblot used an annexin A11 antibody (Abcam #ab236599 at 1:10,000) and a TDP-43 antibody (1D3, 1:200) (Fig. 5).

## Results

### Annexin A11 aggregation in FTLD–TDP Type C

Five rare *ANXA11* genetic variants (MAF < 0.05%) were identified from sequencing data of autopsied cases (*n* = 822) from the CNDR brain bank (Table 1). This included four variants of uncertain significance and one previously described pathogenic variant, *ANXA11* p.G38R. As pathogenic *ANXA11* variants have been shown to exhibit TDP-43 and annexin A11 aggregates in affected brain regions, all five cases were stained for pTDP-43 and annexin A11 using immunohistochemistry. Cases #4 and 5, corresponding to *ANXA11* p.R475W and p.L337H, did not exhibit any pTDP-43 or annexin A11 aggregates. Given that *ANXA11* variants have been associated with a dual TDP-43 proteinopathy and annexinopathy, and because these two cases were clinically and neuropathologically diagnosed with progressive supranuclear palsy, a tauopathy, this suggested that these two variants may not play a significant role in TDP-43 proteinopathy.

**Fig. 1:**
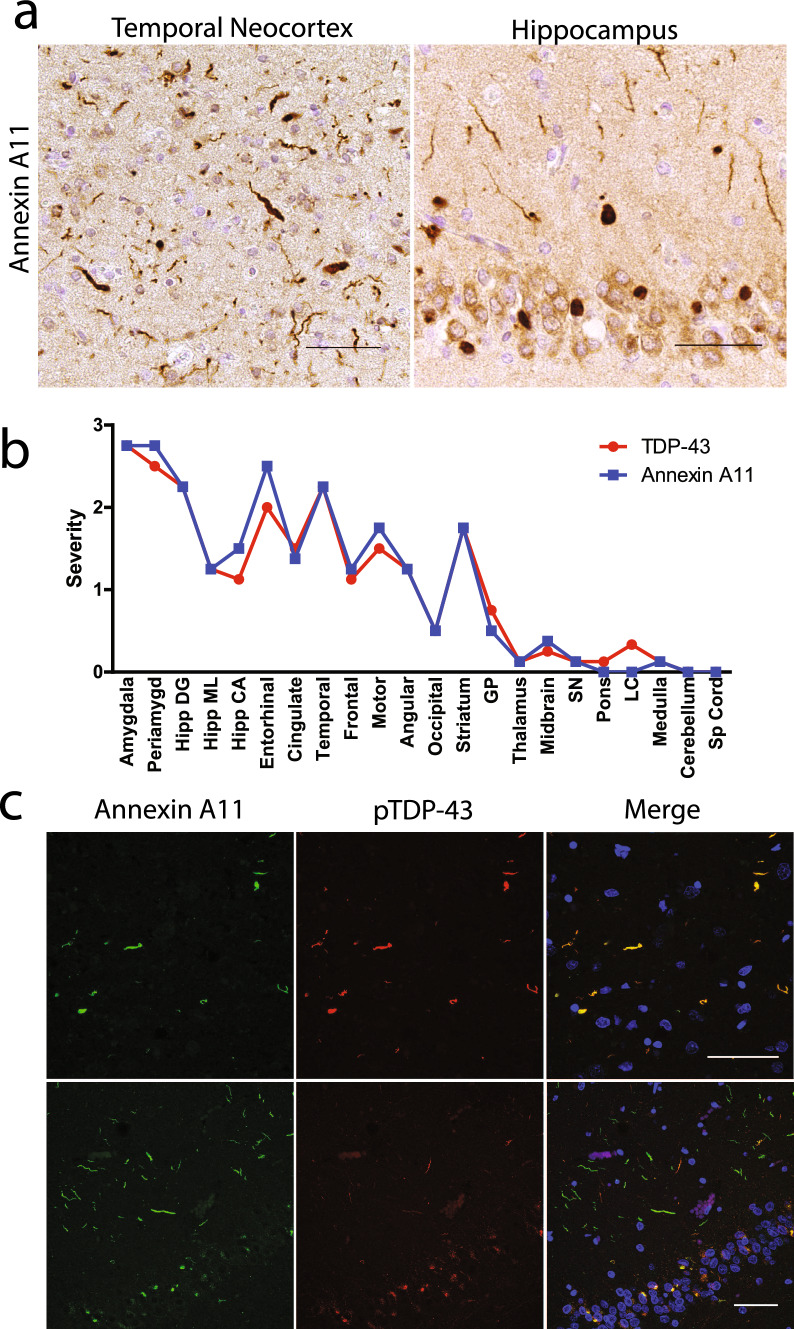
Annexin A11 aggregation in FTLD–TDP Type C. Annexin A11 antibody specifically labels canonical Type C pathology including **a** long DNs in cortical regions and compact NCIs in the dentate gyrus (representative images, cases #16 and 22; *scale bar* = *50 µm*)*.* **b** Distribution and severity of annexin A11 inclusions closely matched the distribution and severity of TDP-43 inclusions in FTLD–TDP Type C cases. Shown is the average semi-quantitative regional burden (severity score) for each pathology from four cases (cases #16, 22, 33, 48). **c** Double immunofluorescence against annexin A11 and phosphorylated TDP-43 (pTDP-43) reveals abundant co-localization of the two proteinopathies in cortex (case #25, upper row). Case #23 showed annexin A11 positive neurites in the molecular layer of the hippocampus that were less prominent by TDP-43 staining (lower row; s*cale bar* = *50 µm*)

Case #3, which harbored an *ANXA11* p.G199S variant, was diagnosed neuropathologically as FTLD–TDP Type C. This case exhibited abundant numbers of annexin A11 aggregates that looked similar to the ropy, DNs characteristic of FTLD–TDP Type C. Since FTLD–TDP Type C is generally sporadic and not associated with known pathogenic genetic variants, we explored whether annexin A11 aggregate pathology could be more broadly associated with various TDP-43 proteinopathies by screening a series of autopsy cases for the presence of annexin A11 inclusions (Supplementary Table 2). This autopsy cohort included all the additional FTLD–TDP Type C cases available in our brain bank (*n* = 34), together with 69 non-Type C FTLD–TDP cases, 75 ALS cases including 5 with pathogenic *SOD1* variants, 130 AD cases with LATE-NC and others. Interestingly, all additional FTLD–TDP Type C cases (*n* = 34/34) were positive for annexin A11 aggregate pathology (Table 2). In these Type C cases, annexin A11 aggregate morphology was identical to that of TDP-43 inclusions (Fig. 1a) characterized by long, thick DNs involving all neocortical layers (Fig. 1a).

**Table 2:** Demographic, genetic and clinicopathological data of annexinopathy cases

Case	Group	Onset	Age	Sex	Clinical diagnosis	Neuropathological diagnosis				Genetics	
						Primary	Secondary	Tertiary	Type	Variant	APOE
1	Vacuolar annexinopathy	65–70	70–75	M	PSP/FTD-NOS	FTLD–UPS	ADNC, low			*ANXA11* p.P75S	E3/E3
2	ALS with annexinopathy	60–65	60–65	M	ALS (Definite)	ALS	PART		B	*ANXA11* p.G38R	E2/E3
3	FTLD–TDP	60–65	65–70	F	svPPA	FTLD–TDP	AGD	ADNC, low	A	*GBA* p.N409S	E3/E3
4	FTLD–TDP	60–65	70–75	F	bvFTD/ALS (Definite)	FTLD–TDP	LBD	PART	B	*C9orf72* expansion	E3/E4
5	FTLD–TDP	71	> 90	M	bvFTD	FTLD–TDP	PART		A		E3/E3
6	ALS	50–55	55–60	M	ALS (Definite)/bvFTD	ALS	FTLD–TDP	ADNC, low	B	*TBK1* p.R308*	E3/E4
7	ALS	70–75	70–75	F	ALS (Probable)	ALS	FTLD–TDP	PART	B	*C9orf72* expansion	E3/E4
8	AD-LATE	58	68	M	AD Probable	ADNC, high	LATE-NC	LBD			E3/E3
9	AD-LATE	60	69	F	AD Probable	ADNC, high	LATE-NC	LBD			n/a
10	AD-LATE	59	72	F	lvPPA	ADNC, high	LATE-NC	LBD			E3/E3
11	AD-LATE	59	74	M	AD Probable	ADNC, high	LATE-NC	LBD			E3/E4
12	AD-LATE	59	75	M	svPPA	ADNC, high	LATE-NC				E3/E3
13	AD-LATE	65	77	F	AD Probable	ADNC, high	LATE-NC	LBD			E3/E4
14	AD-LATE	72	87	F	AD Probable	ADNC, high	LATE-NC	LBD			E3/E4
15	AD-LATE	71	> 90	M	AD Probable	ADNC, high	LATE-NC	LBD			E4/E4
16	FTLD–TDP	45–50	50–55	M	bvFTD	FTLD–TDP			C	*C9orf72* expansion	E2/E2
17	FTLD–TDP	55	61	F	svPPA/bvFTD	FTLD–TDP			C		E2/E3
18	FTLD–TDP	n/a	63	M	svPPA	FTLD–TDP			C		E3/E4
19	FTLD–TDP	54	63	M	svPPA	FTLD–TDP	PART		C		E3/E3
20	FTLD–TDP	58	63	F	bvFTD	FTLD–TDP	ADNC, low		C		E3/E4
21	FTLD–TDP	55	64	M	PPA-NOS	FTLD–TDP			C		E3/E3
22	FTLD–TDP	60	65	M	bvFTD/svPPA	FTLD–TDP	ADNC, low		C		E3/E3
23	FTLD–TDP	52	65	M	bvFTD/svPPA	FTLD–TDP	PSP		C		E2/E3
24	FTLD–TDP	53	66	F	svPPA/bvFTD	FTLD–TDP	ADNC, low		C		E3/E3
25	FTLD–TDP	57	67	F	bvFTD/svPPA	FTLD–TDP	ADNC, low		C		E3/E4
26	FTLD–TDP	62	68	F	svPPA	FTLD–TDP	ADNC, low		C		E3/E3
27	FTLD–TDP	56	68	M	svPPA/bvFTD	FTLD–TDP	PART		C		E2/E3
28	FTLD–TDP	59	69	F	PPA-NOS	FTLD–TDP	Tauopathy	LBD	C		E3/E3
29	FTLD–TDP	63	70	M	svPPA/bvFTD	FTLD–TDP	ADNC, low		C		E3/E3
30	FTLD–TDP	59	70	M	svPPA	FTLD–TDP	ADNC, low		C		E3/E3
31	FTLD–TDP	60	71	M	svPPA	FTLD–TDP	ADNC, low		C		E3/E4
32	FTLD–TDP	62	72	F	svPPA	FTLD–TDP	PART		C		E3/E3
33	FTLD–TDP	68	73	M	bvFTD	FTLD–TDP	ADNC, low	CVD	C		E3/E3
34	FTLD–TDP	65	73	M	svPPA	FTLD–TDP	LBD	PART	C		E2/E3
35	FTLD–TDP	65	73	M	svPPA/bvFTD	FTLD–TDP	PART		C		E3/E3
36	FTLD–TDP	60	74	F	svPPA/bvFTD	FTLD–TDP	PART		C		E3/E3
37	FTLD–TDP	66	74	M	AD Probable	FTLD–TDP	ADNC, low		C		E3/E3
38	FTLD–TDP	67	76	F	svPPA	FTLD–TDP	ADNC, low		C		E3/E4
39	FTLD–TDP	65	76	F	svPPA	FTLD–TDP	CVD		C		E2/E2
40	FTLD–TDP	65	76	M	bvFTD	FTLD–TDP	ADNC, low	CVD	C		E3/E4
41	FTLD–TDP	67	77	M	svPPA/bvFTD	FTLD–TDP	ADNC, low		C		E3/E3
42	FTLD–TDP	55	77	M	bvFTD	FTLD–TDP	PART		C		E3/E3
43	FTLD–TDP	68	79	M	AD Probable	FTLD–TDP	ADNC, low		C		E3/E3
44	FTLD–TDP	67	80	M	AD Probable	FTLD–TDP	ADNC, low		C		E3/E4
45	FTLD–TDP	60–65	80–85	M	bvFTD/AD Probable	FTLD–TDP	ADNC, low		C	*ANXA11* p.G199S	E3/E3
46	FTLD–TDP	79	81	F	PPA-NOS	FTLD–TDP	ADNC, low		C		E2/E3
47	FTLD–TDP	72	82	M	CVD/AD Possible	FTLD–TDP	ADNC, low	CVD	C		E4/E4
48	FTLD–TDP	67	83	M	svPPA/bvFTD	FTLD–TDP	LBD	ADNC, low	C		E3/E3
49	FTLD–TDP	77	83	M	CBS	FTLD–TDP	LBD	CVD	C		E3/E3
50	FTLD–TDP	78	87	F	AD Probable	FTLD–TDP	ADNC, low	LBD	C		E3/E4

*AD* Alzheimer’s disease, *ADNC* Alzheimer’s disease neuropathologic change, *AGD* argyrophilic grain disease, *ALS* Amyotrophic Lateral Sclerosis, *CBS* Corticobasal syndrome, *CVD* cerebrovascular disease, *bvFTD* behavioral variant Frontotemporal degeneration, *FTLD-U* Frontotemporal lobar degeneration with ubiquitin positive inclusions, *LBD* Lewy body disease, *lvPPA* logopenic primary progressive aphasia, *PART* primary age-related tauopathy, *PPA-NOS* Primary progressive aphasia not otherwise specified, *PSP* progressive supranuclear palsy, *svPPA* semantic variant primary progressive aphasia. Age ranges are provided to help maintain anonymity for cases where genetic data (aside from APOE genotype) is reported

To assess the severity and distribution of these pathologies in FTLD–TDP Type C, the annexin A11 and TDP-43 aggregate burden was assessed using a semi-quantitative scale across brain regions for four cases, demonstrating that the burden of annexin A11 and TDP-43 inclusions were highly similar in both amount and distribution (Fig. 1b). For example, annexin A11-positive DNs were frequently more severe in temporal cortex, amygdala, and medial temporal lobe, with compact NCIs are more abundant in both the dentate gyrus and striatum. In some cases with severe dentate gyrus pathology, prominent annexin A11-positive neurites were detected in the hippocampal molecular layer (Fig. 1a). Areas with less TDP-43 proteinopathy, such as occipital cortex or globus pallidus, displayed a concurrent lower level of annexin A11 proteinopathy as well. TDP-43 proteinopathy is rare to non-existent in FTLD–TDP Type C brainstem and spinal cord [9], and this is the case with annexinopathy as well. The concordance between annexin A11 and TDP-43 pathology was further supported by double immunofluorescence which demonstrated strong colocalization between annexin A11 and pTDP-43 in most instances, although occasional aggregates were preferentially labelled with annexin A11 or pTDP-43. For example, one case demonstrated annexin A11 positive neurites in the molecular layer of the hippocampus that were relatively weakly stained for pTDP-43 (Fig. 1c).

FTLD–TDP Type C cases are typically sporadic without a strong family history of disease, and the rare p.G199S *ANXA11* variant was detected in only one Type C case (Table 2). The finding of co-aggregation of annexin A11 and pTDP-43 across all Type C cases suggests that the p.G199S variant is unlikely to be driving annexin A11 aggregate formation. Rather, we provide evidence by morphology, abundance, distribution and colocalization for a strong relationship between annexin A11 and TDP-43 pathology in sporadic FTLD–TDP Type C cases.

### Annexin A11 aggregation in other TDP-43 proteinopathies

We also identified an additional thirteen cases with evidence of annexin A11 pathology including eight AD cases with LATE-NC, two FTLD–TDP Type A cases, one FTLD–TDP Type B case, and two ALS cases (Table 2). Annexin A11 pathology was not found in cases without TDP-43 proteinopathy including FTLD–FUS cases. Annexin A11 pathology was uncommon outside of FTLD–TDP Type C cases with a prevalence of 6% in LATE-NC, 6% in FLTD–TDP Type A, 3% in FTLD–TDP Type B, and 3% in ALS. The annexin A11 positive cases were not clearly distinguished by age of onset, age at death, sex, clinical diagnosis or *APOE* genetic status compared to annexin A11 negative cases. However, seven of the eight (88%) LATE-NC cases also had Lewy body disease (amygdala-predominant) which is higher than that seen in the other AD cases with LATE-NC but without annexinopathy (60%, *n* = 73/122). In addition, several cases with annexin A11 aggregation harbored other known pathogenic genetic variants (Table 2).

**Fig. 2:**
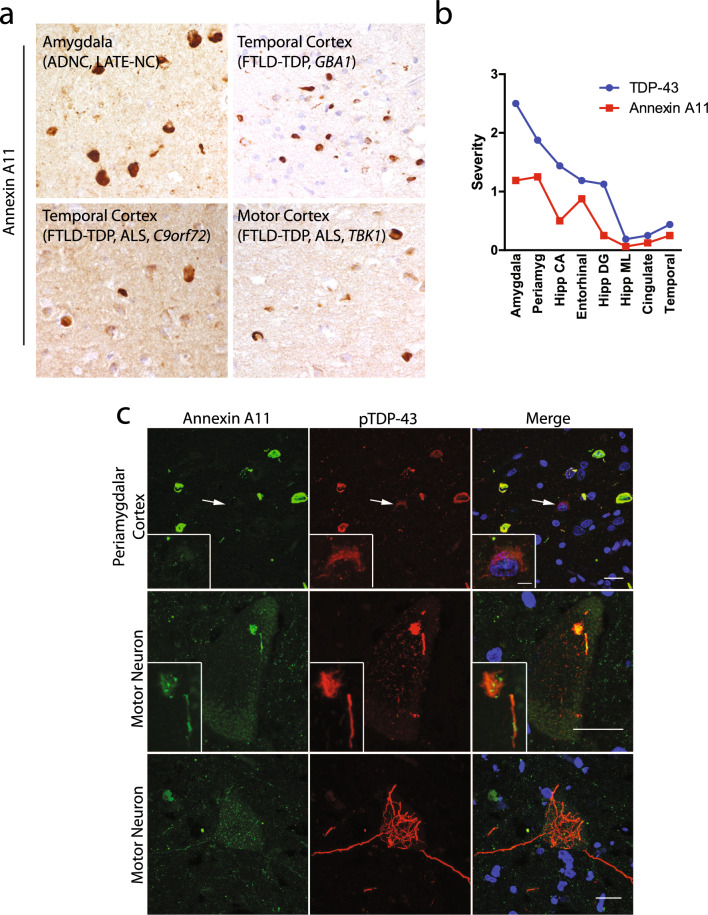
Spectrum of annexin A11 aggregation in TDP-43 proteinopathies. Annexin A11 pathology paralleled the morphology of the TDP-43 inclusions present in each disease. **a** In LATE-NC (upper row, left), annexin A11 inclusions were observed as globular NCIs or small neurites (case #11). In a case of FTLD-TDP Type A with *GBA* p.N409S (upper row, right), there were abundant compact NCIs in the superficial layers of the cortex (case #3). In a Type B case with a *C9orf72* expansion (lower row, left), annexin A11 positive NCIs in superficial and deep layers of the cortex were noted (case #4). In a *TBK1* variant (lower row, right), annexin A11 positive crescent-shaped and ring-like NCIs were apparent (case #6; *scale bar* = *50 µm*)*.* **b** Distribution of annexin A11 inclusions closely matched the distribution of TDP-43 inclusions in LATE-NC but was typically less severe. Shown is the average semi-quantitative regional aggregate burden from all annexin A11 positive LATE-NC cases (cases #8–15). **c** Double immunofluorescence against annexin A11 and TDP-43 reveals co-localization of the two proteinopathies in the cerebrum (case #7, upper row) and occasional comingling within motor neurons (middle row), although motor neuron aggregates in ALS were often TDP-43 positive and annexin A11 negative (lower row). S*cale bar* = *20 µm, insert 5 µm*

Annexin A11 aggregates typically matched the different morphologies and distributions of TDP-43 inclusions in these non-Type C cases (Fig. 2a). For example, in LATE-NC cases, annexin A11 formed globular NCIs primarily confined to the medial temporal lobe including amygdala and hippocampus. Indeed, one LATE-NC case (Table 2, case #12) for which bilateral hippocampus regions were available, both TDP-43 and annexin A11 aggregates were present in the left hippocampus and neither pathology was present in the right hippocampus. TDP-43 inclusions in a case of FTLD–TDP Type A with a *GBA1* risk variant were comprised of compact NCIs in the superficial layers of the cortex which matched the morphology and distribution of annexin A11 inclusions. A case of FTLD–TDP Type B with ALS harboring a *C9orf72* repeat expansion exhibited TDP-43 and annexin A11 positive NCIs in superficial and deep layers of the neocortex. A case of ALS with FTLD–TDP harboring a *TBK1* pathogenic variant exhibited TDP-43 and annexin A11-positive inclusions comprised of crescent-shaped and ring-like NCIs and short DNs [6].

In non-Type C cases with annexinopathy, TDP-43 aggregates appeared to be more prominent than annexin A11 aggregates. In the eight LATE-NC cases that exhibited annexin A11 pathology (Table 2, cases #8–15), semi-quantitative grading demonstrated that TDP-43 and annexin A11 inclusions followed a similar distribution but with a lower burden of annexin A11 aggregates compared to TDP aggregates (Fig. 2b).

When present in motor neuron disease, TDP-43 inclusions were more common than annexin A11 inclusions. In the two ALS cases (Table 2, cases #6 and 7) annexin A11 and TDP-43 aggregates were present in upper and lower motor neurons, striatum, amygdala and hippocampus, and frontal and temporal lobe regions. However, TDP-43 aggregates were present in thalamus (cases #6 and 7), and pons and medulla (case #7) where annexin A11 aggregates were difficult to find. In the FTLD–TDP case with a secondary clinical diagnosis of ALS (Table 2, case #4), annexin A11 aggregates were confined to both upper and lower motor neurons, and amygdala and hippocampus, while TDP-43 pathology was more widespread and included other frontal and temporal lobe, striatum and thalamus, and substantia nigra and medulla areas. Indeed, double immunofluorescence of spinal cord (Fig. 2c) showed partial intermingling of annexin A11 and TDP-43 within a skein-like inclusion without strong signal within the more delicate TDP-43 aggregate structures (Fig. 2c, middle row), in addition to several annexin A11 negative but TDP-43 positive motor neuron inclusions (Fig. 2c, lower row). Thus, when annexin A11 aggregates are present in non-Type C TDP-43 proteinopathies, its overall distribution matches the underlying TDP-43 proteinopathy in terms of morphology but with generally reduced distribution and severity.

### Neuropathology of ANXA11 variant cases

**Fig. 3:**
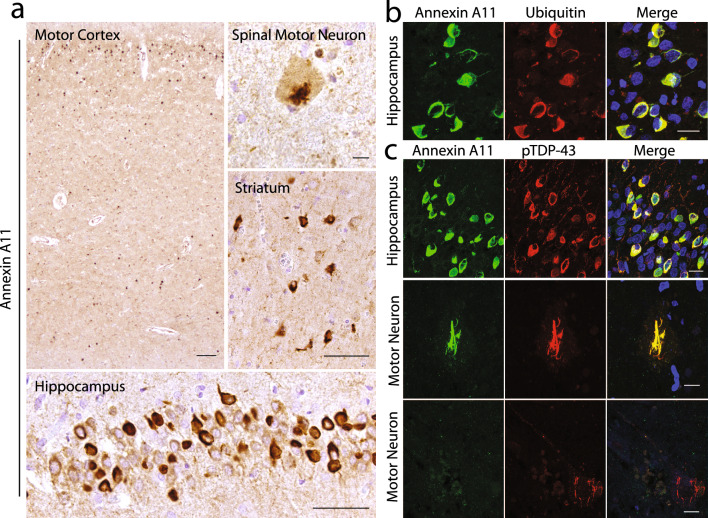
Annexin A11 pathology in ALS due to *ANXA11* p.G38R. **a** Annexin A11 positive inclusions were moderate to severe in amount, comprised of NCIs in the motor cortex (*scale bar* = *100 µm)*, hippocampus and striatum (*scale bar* = *50 µm)*, with rare skein-like spinal motor neuron inclusions (*scale bar* = *10 µm)*. **b** By double immunofluorescence, annexin A11 pathology was typically ubiquitin positive (*scale bar* = *20 µm*)*.* **c** Many annexin A11 aggregates appeared to be TDP-43 positive such as NCIs in the dentate gryus (upper row, *scale bar* = *20 µm*)*.* Rare annexin A11 positive spinal motor neuron inclusions were observed that were TDP-43 positive (middle row, *scale bar* = *10 µm*), but many spinal motor neuron inclusions were TDP-43 positive and annexin A11 negative (lower row, *scale bar* = *20 µm*)

Returning to the results of the genetic screen, we identified one ALS case (Table 2, Case 2) with *ANXA11* p.G38R that has already been reported to be associated with familial ALS. Clinically, this 65-year-old male presented with bulbar onset motor weakness consistent with ALS that progressed over an approximately 2-year course without cognitive impairment. There was no documented family history of motor neuron disease, although one parent was reported to have a slight tremor. Neuropathologically, the major findings were moderate loss of anterior horn motor neurons and long tract degeneration within the spinal cord. TDP-43 positive inclusions were present in residual neurons of the motor cortex and spinal cord, in addition to a moderate to severe burden of inclusions in the medial temporal lobe, frontal and temporal neocortex, and striatum. Neurofibrillary tangles (Braak stage III) were also present. Beta-amyloid plaques and Lewy bodies were not identified. Notably, annexin A11 positive inclusions were found in all brain areas where TDP-43 inclusions were found (Fig. 3). These included abundant compact NCIs in the motor cortex, hippocampus, and striatum (Fig. 3a). Skein-like inclusions were rare. As expected, annexin A11 aggregates were ubiquitinated as demonstrated by double immunofluorescence (Fig. 3b). Moreover, there was considerable co-localization between TDP-43 and annexin A11 (Fig. 3c) except for the spinal cord where both annexin A11 and TDP-43 double positive (middle row), and TDP-43 only motor neuron aggregates were observed (lower row).

**Fig. 4:**
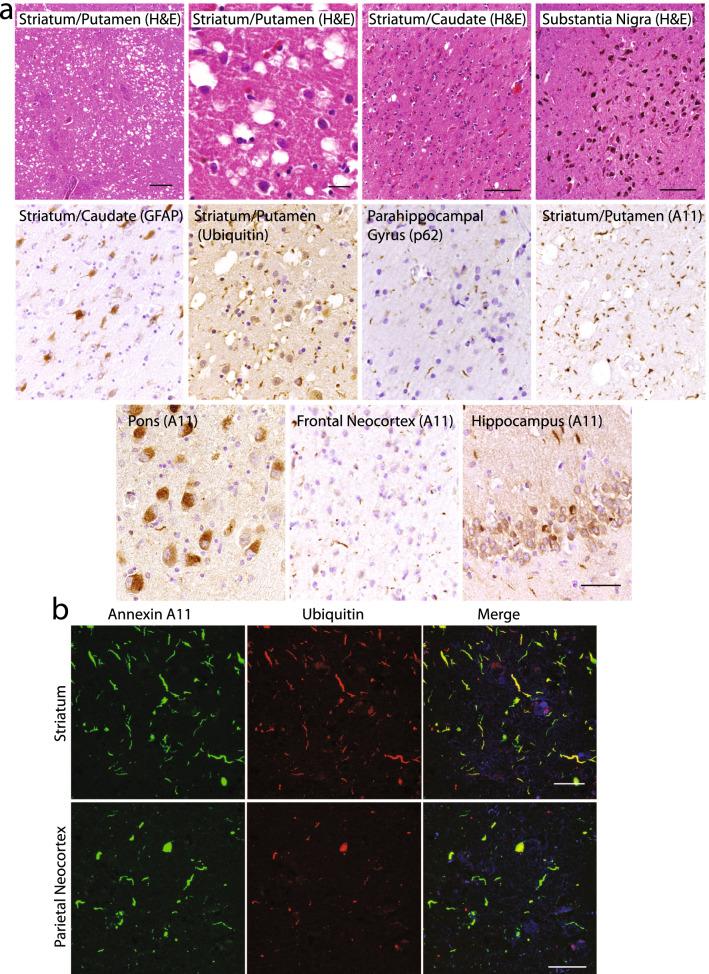
Vacuolar annexinopathy in FTLD-U due to *ANXA11* p.P75S. **a** H&E stained sections revealed severe striatal degeneration with prominent vacuolization of the putamen (upper row, *scale bars* = *200, 50 and 100 µm, respectively)*, but with preservation of the substantia nigra (*scale bar* = *200 µm*). GFAP stain highlights reactive astrocytosis. Ubiquitin and p62 immunohistochemistry revealed abundant neuritic inclusions (middle row). Annexin A11 aggregates affected many regions of the brain, including a high burden of dystrophic neurites in striatum (middle row), globular NCIs in the pons, dystrophic neurites in neocortex, and both dystrophic neurites and NCIs in the hippocampus (lower row, *scale bar* = *50 µm*). **b** Double immunofluorescence showed striatal annexin A11 aggregates to be strongly ubiquitin positive (upper row) compared to neocortical regions (parietal cortex, lower row; *scale bar* = *50 µm*)

Finally, a novel *ANXA11* p.P75S variant was identified in a patient who presented with cognitive and behavioral difficulties including emotional incontinence, progressive non-fluent speech and language difficulties along with executive dysfunction, parkinsonism with postural instability and an occulomotility disorder of hypometric saccades with square wave jerks and eyelid apraxia, clinically consistent with a form of progressive supranuclear palsy (PSP) (Table 2, case #1). One of the decedent’s parents died in their 40s due to syphilis while the other parent died in their 90s due to congestive heart failure. Neuropathologically, the major finding was severe degeneration and gliosis of the striatum including a marked, prion disease-like vacuolization of the putamen (Fig. 4a). This was accompanied by a severe burden of p62 and ubiquitin positive inclusions, most notably consisting of neuritic inclusions in the striatum and NCIs affecting the dentate gyrus of the hippocampus. Degeneration of neocortical areas was inconspicuous, although neocortex did exhibit a moderate burden of p62 and ubiquitin positive neuritic inclusions. Grossly, the substantia nigra was well pigmented consistent with microscopic examination which did not reveal nigral degeneration. A low density of neurofibrillary tangles (Braak stage I) and rare diffuse β-amyloid plaques were also present, indicative of a low level of AD neuropathologic change. Other proteinopathies, including TDP-43 positive inclusions were absent.

Remarkably, annexin A11 immunohistochemistry revealed abundant annexinopathy in most regions of the brain save the cerebellum (Fig. 4a, lower row). Morphologically, annexin A11 aggregates were most severe in the striatum, consisting of primarily neuritic inclusions. Examination of the pons revealed unusual, jagged NCIs involving the nuclei of the basis pontis. Indeed, most regions of the brain were affected including a moderate burden of neuritic inclusions involving neocortical and limbic (amygdala and hippocampus) regions. The hippocampus displayed a moderate burden of NCIs within the dentate gyrus and neuritic inclusions in the molecular layer. Interestingly, double immunofluorescence reveals that most of the annexin A11 aggregates in the striatum were ubiquitinated while areas with fewer annexin A11 aggregates such as the anterior cingulate and parietal (angular) neocortex showed only partial colocalization with ubiquitin (Fig. 4b). This suggests that annexin A11 is a more sensitive marker of pathology than ubiquitin and that the striatal involvement in this case is more mature, consistent with the clinical PSP-like phenotype. We suggest that annexin A11 aggregation together with the marked vacuolization and degeneration of the striatum are the most striking findings in this case, raising the possibility that annexin A11 dysfunction may be driving striatal degeneration. This unique case of vacuolar annexinopathy implicates annexin A11 dysfunction as being sufficient to cause neurodegeneration independent of TDP-43 proteinopathy.

### Biochemical characterization of annexinopathies

**Fig. 5:**
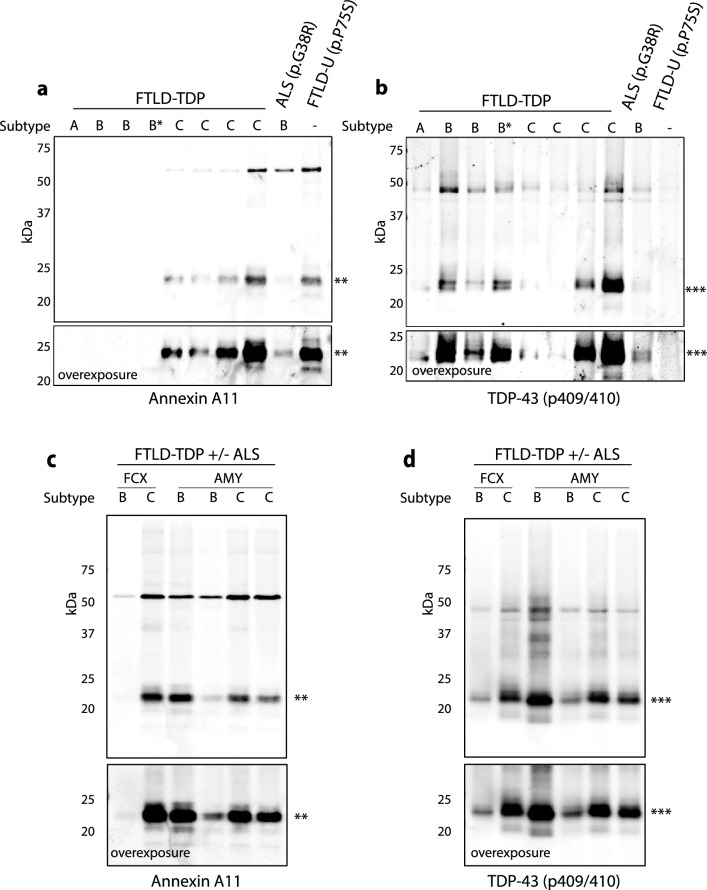
Biochemical analyses of annexin A11 and TDP-43 in FTLD–TDP and *ANXA11* variant cases. Immunoblots against annexin A11 and TDP-43 of sarkosyl-insoluble fractions from ALS, FTLD–TDP and *ANXA11* variant cases. For panels (**a**) and (**b**), lane 1 is an FTLD–TDP Type A case without annexinopathy, lanes 2–4 are FTLD–TDP Type B cases without annexinopathy where the asterisk denotes a case with concurrent ALS, and lanes 5–10 correspond to annexinopathy cases in this study (cases #26, 43, 18, 17, 2 & 1, respectively). **a** Annexin A11 positive bands were detected in all Type C cases and both *ANXA11* variant cases at ~ 56 kDa and between 20 and 25 kDa (**), that were not detected in FTLD–TDP Type A or Type B cases without annexinopathy. **b** All FTLD–TDP cases and the ALS p.G38R variant show phosphorylated full-length TDP-43-positive bands at ~ 43 kDa and truncated TDP-43 between ~ 23 and 25 kDa (***). Truncated TDP-43 was not detected in the *ANXA11* p.P75S variant case. For panels (**c**) and (**d**), lanes 1 and 2 are frontal cortex (FCX) while lanes 3 to 6 are amygdalar region (AMY) where lanes 1 to 6 correspond to cases #4, 17, 6, 7, 41 and 19, respectively. **c** Annexin A11 positive bands at ~ 56 kDa and between 20 and 25 kDa (**) were detected in all cases. **d** Same fractions were also positive for phosphorylated full-length TDP-43 at ~ 43 kDa and truncated TDP-43 between ~ 23 and 25 kDa (***)

To determine whether the annexin A11 aggregates seen histologically were associated with accumulation of insoluble and/or modified annexin A11 protein, sequential biochemical extraction of brain tissue was performed to isolate soluble and sarkosyl-insoluble proteins. A series of FTLD–TDP cases corresponding to cases without annexin A11 aggregates (FTLD–TDP Types A and B), and cases with annexin A11 aggregates (FTLD–TDP Type C) were assessed in addition to cases with *ANXA11* p.G38R and p.P75S variants. Full length annexin A11 was seen in all brain tissues within the soluble fraction (Supplementary Fig. 1a). In contrast, insoluble full-length annexin A11 was detected in all four FTLD–TDP Type C cases and both *ANXA11* variant cases but was absent in cases of FTLD–TDP Type A and B (Fig. 5a). Moreover, a ~ 23 kDa annexin A11 fragment was seen in the FTLD–TDP Type C and both *ANXA11* variant cases but was absent in FTLD–TDP Type A and B cases (Fig. 5a). This annexin A11 fragment was detected using two different annexin A11 antibodies including an antibody that recognizes an epitope within the C-terminal amino acid residues 250 to 505 (Supplementary Fig. 1b) and an antibody that recognizes an epitope within the N-terminal amino acid residues 1 to 180 (Supplementary Fig. 1c). Based on the ~ 23 kDa size of the annexin A11 fragment, corresponding to roughly 209 amino acids, the detection of this truncated protein by both N-terminal and C-terminal annexin A11 antibodies suggests that the fragment is cleaved at both the N- and C-termini. Finally, TDP-43 immunoblot of sarkosyl-insoluble fractions revealed the accumulation of pathologic C-terminal TDP-43 fragments in all FTLD–TDP cases and the *ANXA11* p.G38R case (Fig. 5b). The vacuolar annexinopathy case with *ANXA11* p.P75S had no detectable C-terminal TDP-43 fragment, consistent with the absence of TDP-43 aggregates by immunohistochemistry (Fig. 5b).

FTLD–TDP Type B cases with annexinopathy were also studied for the accumulation of insoluble and truncated annexin A11. Frontal cortex or amygdalar region (amgydala with periamygdalar cortex) from FTLD–TDP Type B (Table 2, case #4) or two FTLD–TDP Type B with ALS cases (Table 2, #6–7) were compared with FTLD–TDP Type C cases (Table 2, cases #17, 19 and 41). In all cases, both full length and truncated annexin A11 was apparent (Fig. 5c). Insoluble pTDP-43 including truncated pTDP-43 was also detected in these cases (Fig. 5d). Frozen tissue from LATE-NC or FTLD–TDP Type A cases with annexinopathy were not available for biochemical analysis.

Finally, to check if annexin A11 aggregates formed amyloid, we performed thioflavin S histochemistry on tissue from the p.G38R and p.P75S variants and a subset of FTLD–TDP Type C cases which was negative in all cases. We also performed immunohistochemistry to see if any FUS positive or G3BP1 positive inclusions were present which was negative for FUS proteinopathy or G3BP1-positive aggregates.

## Discussion

This study identifies annexin A11 aggregation as a consistent feature of FTLD–TDP Type C, and the presence of annexin A11 aggregates in a subset of other TDP-43 proteinopathy neurodegenerative diseases. While all FTLD–TDP Type C cases were characterized by the accumulation of annexin A11 aggregates, only ~ 3 to 6% of other TDP-43 proteinopathy cases exhibited annexinopathy. Notably, this autopsy cohort was recruited from tertiary medical center, and so referral bias may have influenced the prevalence of annexinopathy across the spectrum of TDP-43 proteinopathy cases.

The presence of annexin A11 aggregation in FTLD–TDP Type C and rare *ANXA11* variant cases coincided with the presence of sarkosyl-insoluble full length and fragmented annexin A11 protein. The insoluble annexin A11 fragment was similar across these cases which raises the possibility that similar aggregates are forming in sporadic and genetic forms of annexinopathy. In addition, in FTLD–TDP Type C, annexin A11 inclusions are morphologically similar to and colocalize with TDP-43 aggregates, indicating that both pathologies may share upstream pathogenic mechanisms. Notably, the atomic structure of TDP-43 aggregates in FTLD–TDP Type C cases has not been solved, perhaps due to our ignorance regarding the presence of annexin A11 aggregates in these cases.

Multiple aggregating misfolded proteins are a key characteristic of neurodegenerative disease. For example, AD with Lewy bodies and/or LATE-NC is more prevalent than pure AD [8, 21]. When considering the 3–6% of TDP-43 proteinopathy cases with annexin A11 aggregates (i.e., cases without FTLD–TDP Type C and without a pathogenic *ANXA11* variant), there is a suggestion that increased proteostatic burden or genetics may be involved. Indeed, nearly every case of LATE-NC with annexinopathy in this autopsy cohort exhibited co-accumulation of beta-amyloid, tau, alpha-synuclein, and TDP-43. Moreover, most other cases of TDP-43 proteinopathy with annexinopathy harbored pathogenic or strong risk variants in other genes (*C9orf72, GRN, TBK1*, or *GBA1)*. We speculate that annexinopathy is enriched in cases with a high burden of proteinopathy and/or genetic predisposition.

Several pathogenic *ANXA11* variants have been described across the entire protein. The vast majority of cases present with ALS, but frontotemporal dementia, progressive supranuclear palsy, inclusion body myopathy, and other clinical syndromes have also been reported [29]. Very few cases have been autopsied. For those with autopsies, the p.D40G variant has been described to exhibit annexin A11 aggregates that are morphologically distinct from TDP-43 inclusions, often with little colocalization [25]. In contrast, a p.G38R variant case was noted to have TDP-43 and annexin A11 aggregates comingling within the same neurons when using dual immunohistochemistry [26]. We describe here similar findings associated with the p.G38R variant where there was a close relationship between annexin A11 and TDP-43 aggregates which often, but not always, colocalized with each other when using immunofluorescence. We also note that the p.G38R variant was associated with the accumulation of full length and fragmented annexin A11 protein, similar to other annexinopathy cases (Fig. 5a), but with perhaps less truncated protein. A splice variant, c.1086 + 1G > A, has also been described to be associated with ALS with both TDP-43 proteinopathy and annexinopathy that partially overlap and colocalize [23]. Finally, we describe here a novel *ANXA11* variant, p.P75S, which we propose is pathogenic based on its absence in normal populations and the presence of annexinopathy, confirmed both pathologically and biochemically. This case exhibited profound vacuolization and degeneration of the striatum, consistent with the clinical history of parkinsonism. Moreover, this variant exhibited annexinopathy in the absence of TDP-43 proteinopathy. This unique case of vacuolar annexinopathy suggests that annexin A11 dysfunction may be sufficient to cause neurodegeneration.

Annexin A11 and TDP-43 belong to a class of proteins with low-complexity domains that facilitate the formation of condensates via relatively weak, multivalent interactions which result in liquid–liquid phase separation [11]. Indeed, proteomics studies suggest that there is no strong or stable protein–protein interaction between annexin A11 and TDP-43 [5, 12]. Annexin A11 has been established to function as a tether between lysosomes and RNP granules [14]. Annexin exhibits an N-terminal low complexity domain which is needed for interaction with RNP granules, and a C-terminal annexin repeat domains which facilitates phospholipid binding in a calcium-dependent manner. Thus, as lysosomes are trafficked down the axon by kinesin, annexin A11 facilitates the tethering of RNP transport granules to lysosomes dependent on both N-terminal and C-terminal domains, allowing the RNP granules to hitchhike along the axon. Interestingly, our biochemical analysis suggests that pathologic annexin A11 protein is both N- and C-terminally truncated. While this may suggest that aggregated annexin A11 may not be functional, we did not observe changes in the amount of normal soluble annexin A11 in these cases. Thus, it is not clear whether annexin A11 aggregation results in a loss of normal annexin A11 function. In contrast, the accumulation of axonal protein aggregates likely disrupts normal axonal transport pathways. Indeed, annexin A11 aggregate formation and neurodegeneration appear to be highly correlated in this autopsy cohort, raising the possibility that annexin A11 aggregation results in a toxic gain of function.

Pathogenic *TARDBP* variants increase the viscosity of liquid-like RNP granules, resulting in impaired axonal transport [4, 15]. Similarly, pathogenic *ANXA11* variants impair axonal transport due to impaired ability of annexin A11 to function as a molecular tether of RNP granules to lysosomes [14]. Indeed, different *ANXA11* variants appear to have different downstream effects including altered cofactor binding, altered calcium responsiveness, and altered phase separation leading to an increased propensity to aggregate [17, 24, 25]. Notably, there is a predominance of neuritic inclusions in both FTLD–TDP Type C and vacuolar annexinopathy which we speculate may be related to the normal axonal localization of annexin A11 protein. Together, these findings reveal the heterogeneity of annexinopathy across diverse TDP-43 proteinopathies including the co-accumulation of TDP-43 and annexin A11 in all cases of FTLD–TDP Type C examined here, while providing evidence that a primary annexinopathy may be sufficient to cause neurodegeneration. Further studies are required to better understand the pathophysiologic mechanisms by which TDP-43 and annexin A11 aggregation leads to neurologic disease.

### Supplementary Information

Below is the link to the electronic supplementary material.

Supplementary file1 (DOCX 18 KB)

Supplementary file2 (EPS 3087 KB) Supplementary Figure 1 Immunoblot against annexin A11 of HS-Tx soluble versus sarkosyl-insoluble fractions from FTLD-TDP cases. Lane 1 is an FTLD-TDP Type A case, lanes 2-4 are FTLD-TDP Type B cases, lanes 5-8 correspond to FTLD-TDP Type C cases (Table 2 cases 17, 18, 43 and 26), and lane 9 is RIPA soluble extract from QBI293 cells (iGFP-NLSm). a. Approximately equal levels of soluble annexin A11 protein were observed (Abcam #ab236599). b-c Annexin A11 bands are detected in sarkosyl-insoluble fractions using (b) a C-terminal annexin A11 antibody (Abcam #ab236599) and (c) a N-terminal annexin A11 antibody (Proteintech #1A3C4). Both immunoblots detect the full length at ~56 kDa and fragment between 20-25 kDa in all FTLD-TDP Type C cases but not FTLD-TDP Type A or Type B cases

## Data Availability

All data is presented in the manuscript and/or supplementary materials.
