# Supplementary material for: Annexin A11 aggregation in FTLD–TDP type C and related neurodegenerative disease proteinopathies
Source: Acta Neuropathol. 2024 Jun 19;147(1):104. doi: 10.1007/s00401-024-02753-7 (PMC11186923; doi:10.1007/s00401-024-02753-7)
Supplement: Supplementary file 1 — Supplementary file1 (DOCX 18 KB) [file 401_2024_2753_MOESM1_ESM.docx]

**Supplementary Material**

**Table 1. *ANXA11* Sequencing Screen**

| **Neuropathologic Diagnosis** | **n** |
| --- | --- |
| AD | 194 |
| AD-LATE | 98 |
| ALS | 143 |
| CBD | 47 |
| FTLD-FUS | 4 |
| FTLD-TDP | 94 |
| FTLD-TDP/ALS | 26 |
| FTLD-UPS | 1 |
| PSP | 110 |
| Other | 105 |
| Total | 822 |

**Table 2. Annexin A11 Immunohistochemistry Screen**

| **Neuropathologic Diagnosis** | **n** | **TDP-43+ (n)** | **ANXA11+ (n)** | **ANXA11+ (%)** |
| --- | --- | --- | --- | --- |
| FTLD-TDP Type A | 34 | 34 | 2 | 6% |
| FTLD-TDP Type B | 29 | 29 | 1 | 3% |
| FTLD-TDP Type C | 34 | 34 | 34 | 100% |
| FTLD-TDP Type C with *ANXA11* p.G199S | 1 | 1 | 1 | 100% |
| FTLD-TDP Type E | 6 | 6 | 0 | 0% |
| ALS | 70 | 70 | 2 | 3% |
| *SOD1* ALS | 5 | 0 | 0 | 0% |
| ALS with *ANXA11* p.G38R | 1 | 1 | 1 | 100% |
| ADNC (High) | 131 | 130 | 8 | 6% |
| CBD | 12 | 11 | 0 | 0% |
| FTLD-FUS | 4 | 0 | 0 | 0% |
| LBD/LATE-NC | 22 | 22 | 0 | 0% |
| LATE-NC | 6 | 5 | 0 | 0% |
| PSP | 5 | 5 | 0 | 0% |
| PSP with *ANXA11* p.R475W | 1 | 0 | 0 | 0% |
| PSP with *ANXA11* p.L337H | 1 | 0 | 0 | 0% |
| Vacuolar annexinopathy with *ANXA11* p.P75S | 1 | 1 | 1 | 100% |
| ADNC (Low) | 3 | 1 | 0 | 0% |
| PART | 1 | 0 | 0 | 0% |
| Unremarkable | 1 | 0 | 0 | 0% |
| Total | 368 |  |  |  |
